# Rapid diagnosis of nitrogen status in rice based on Fourier transform infrared photoacoustic spectroscopy (FTIR-PAS)

**DOI:** 10.1186/s13007-019-0482-0

**Published:** 2019-08-19

**Authors:** Ke Wu, Changwen Du, Fei Ma, Yazhen Shen, Dong Liang, Jianmin Zhou

**Affiliations:** 1 The State Key Laboratory of Soil and Sustainable Agriculture, Institute of Soil Science, Chinese Academy of Sciences, Nanjing, 210008 China; 2 University of Chinese Academy of Sciences, Beijing, 100049 China

**Keywords:** FTIR-PAS, Rice leaves, Amides, Nitrogen status diagnosis

## Abstract

**Background:**

An effective and expeditious approach to assess plant nitrogen status is urgently needed in rice production and management as the conventional chemical methods are laborious and time-consuming.

**Results:**

Fourier transform infrared photoacoustic spectroscopy (FTIR-PAS) was used to record the spectra of rice leaves for the effective diagnosis of nitrogen nutrition status. The band in the wavenumber range of 1680 to 1630 cm^−1^ was associated with amide I and that from 1570 to 1510 cm^−1^ with amide II. We attempted to use this information to characterize the nitrogen status in rice plants at different growth stages. The ratio of photoacoustic intensity of amide II to amide I was measured and applied as nitrogen status index, and considering the yields, the ratio showed a positive linear correlation (R^2^ = 0.9) with the total nitrogen of rice leaves. The ratio at the tillering and full panicle stages were more suitable for diagnosis, a ratio of 0.4–0.55 indicated an adequate nitrogen status, ratios lower than 0.4 indicated a poor nitrogen status; whereas ratios greater than 0.55 indicated excessive nitrogen supply.

**Conclusion:**

Our study provides an effective and rapid strategy for nitrogen-supply assessment in rice based on FTIR-PAS, which can guide rational fertilization in rice production.

**Electronic supplementary material:**

The online version of this article (10.1186/s13007-019-0482-0) contains supplementary material, which is available to authorized users.

## Background

Rice is one of the most common staple food crops in eastern Asia and feeds more than 65% of the population in China [1]. Nitrogen plays an important role in crop production and has been a critical factor in rice yields and agricultural environments [2, 3]. However, excessive usage of nitrogen fertilizer in rice cultivation is relatively common, which not only increases the production cost but also pollutes the environment [4, 5]. Therefore, timely and effective measurement of crop nitrogen status is critical for increasing nitrogen use efficiency and reducing environmental degradation [6, 7].

Conventional diagnosis methods currently used for measuring nitrogen status in crops are chemical in nature, which are laborious and time-consuming [8, 9]; thus, chemical analysis is not an effective method for efficient crop fertilization management, especially in precision agricultural management. Therefore, an effective and rapid approach to diagnose plant nitrogen status is urgently needed in rice production and fertilization management.

Modern remote sensing technology has been widely used in the estimation of crop biochemical components, including nitrogen [10, 11]. Most of the remote sensing techniques depend on near-infrared wavelengths. Nowadays, as a promising alternative, Fourier transform infrared photoacoustic spectroscopy (FTIR-PAS) has been effectively applied in many fields, such as analyzing soil physical and chemical properties [12, 13], identification of plant diseases [14], gas monitoring [15], and food safety [16, 17]. Compared to the near-infrared spectrum, the mid-infrared spectrum provides more information about the measured targets [18–20]. From the scattering of plant tissue [21], photoacoustic spectroscopy can capture more information from leaves due to the depth-profiling abilities, and then assess the nutritional status of plants tissue [18]. However, the scope of infrared photoacoustic spectroscopy to assess nitrogen nutrition levels in rice production remains unexplored.

In rice, the accumulation and distribution of nitrogen in vegetative organs and reproductive organs is an important factor in determining yield. The key period for rice to absorb nitrogen is from tillering to flowering stage, and the absorbed nitrogen is mainly stored in the leaves [22]. At the filling stage, the grain requires a large amount of nitrogen. The amount of nitrogen absorbed by the plant is much smaller than the amount of nitrogen accumulated in the mature grain, and most of the grain nitrogen is transferred from the vegetative organs, especially the leaves [23]. It was reported that a major proportion of nitrogen was redistributed from vegetative organs to panicles at grain filling stage, of which 64% was from leaf blades, 16% from leaf sheaths and 20% from stems [24]. Norman reported that at least 40 kg of N per hectare of soil was absorbed between flowering and maturity. The nitrogen content of vegetative parts decreased significantly during flowering and maturity, suggesting that the active nitrogen had been transported to the grains [25]. Considering that the leaf was the main storage organ for nitrogen, and about 65% of the above-ground nitrogen was located in the green leaf blades [26], thus, the nitrogen content of the leaves can be used to assess the nitrogen nutrition status in rice.

Amides are major chemical structures for nitrogen storage in plants [27, 28], and are classified as either amide I or amide II. The amide I band occurred in the range of 1680 to 1630 cm^−1^ mainly because of the C=O vibrational stretching [29], while the amide II band lies within the range of 1570 to 1510 cm^−1^ mainly corresponded to the C–N stretching vibrations and N–H deformation vibrations. In plants, normal nitrogen metabolism initially involves conversion of absorbed nitrates into ammonium ions (NH_4_^+^), which are then used to synthesize amino acids and other macromolecules. Nevertheless, even low concentrations of NH_4_^+^ in plants can be very harmful. Hence, plant tissues synthesize glutamine to utilize any NH_4_^+^ present, thereby, preventing ammonium ion toxicity through glutamate synthase activity. Related research shows that the primary and secondary amides are the dominant forms of nitrogen in plants [27]. Further, an equilibrium between amide II and amide I is reached at a specific nitrogen status. This equilibrium might be altered through transformation between amide I and amide II when the nitrogen supply status changes, thus providing a potential tool for the diagnosis of plant nitrogen status. In this study, we conducted a 4-year field experiment to establish a method involving amide I and amide II of rice leaves based on FTIR-PAS, which can be used for rapid monitoring of the nitrogen status in rice plants.

## Methods

### Experimental locations

Two field experiments were conducted from 2015 to 2018 at the Tangquan (field experiment 1) and Jiangning (field experiment 2) experimental stations of the Nanjing Institute of Soil Science, Chinese Academy of Sciences, Jiangsu province, China (N 32°04′15″, E 118°28′21″ and N 31°48′23.62″, E 118°55′54.69″, respectively). The soil types in the two study areas were paddy soil and river silt soil, and the basic soil properties are as following. The soil in experiment 1 (Tangquan): pH 6.0, soil organic matter 22.26 g kg^−1^, total N 1.31 g kg^−1^, Olsen-P 15.41 mg kg^−1^, NH_4_OAc-K 146.4 mg kg^−1^; the soil in experiment 2 (Jiangning): pH 6.8, soil organic matter 24.65 g kg^−1^, total N 1.72 g kg^−1^, Olsen-P 19.50 mg kg^−1^, NH_4_OAc-K 128.5 mg kg^−1^. Fertilizers used for these two experiments were as follows: compound fertilizer (N–P_2_O_5_–K_2_O:16–8–18), coated urea (N–42%, ISSAS, China), and urea (N–46%) controlled release fertilizer (N–P_2_O_5_–K_2_O, 30–10–12, ISSAS, China), and urea (N–46%).

### Elemental design

Three fertilizer treatments were applied at the Tangquan experimental station from 2015 to 2018 (Table 1). (1) CO: control with no fertilizer application; (2) CF (Conventional Fertilization): conventional nitrogen fertilization, N (240 kg ha^−1^), P_2_O_5_ (60 kg ha^−1^), and K_2_O (120 kg ha^−1^) (phosphate and potash fertilizers were used as the basal fertilizers; Nitrogen fertilizer was applied as basal fertilizer, tillering fertilizer and panicle fertilizer with the ratio of 5:2:3); and (3) OF (Optimized Fertilization): mixed application of compound fertilizer; urea and coated urea (slow-release nitrogen accounted for 30% of total nitrogen applied), and the application rates were set for N, P_2_O_5_, and K_2_O as that of CF. All fertilizers were applied in artificial broadcasting. Every treatment was designed with four replicates. Each replicate area was 40 m^2^ (4 m × 10 m).

**Table 1:** Cultivars, treatments and fertilizer rate at each station in the experimental years

Experimental station	Seasons	Cultivars	Treatments	Fertilizer rate (kg ha^−1^)		
				N	P_2_O_5_	K_2_O
Tangquan			CO	–	–	–
	2015	Nanjing 46	CF	240	60	120
			OF	240	60	120
			CO	–	–	–
	2016	Nanjing 46	CF	240	60	120
			OF	240	60	120
			CO	–	–	–
	2017	Nanjing 46	CF	240	60	120
			OF	240	60	120
			CO	–	–	–
	2018	Wuyunjing 23	CF	240	60	120
			OF	240	60	120
Jiangning			CO	–	–	–
	2018	Nanjing 5055	CF	255	64	76.5
			OF	192	64	76.5

In 2015, 2016, and 2017, we used the rice cultivar Nanjing 46, a translucent endosperm japonica rice variety with good taste, high production and resistance to rice stripe disease, and in 2018, the cultivar was Wuyunjing 23, a lodging resistant japonica rice cultivar [30, 31]. The 35-day-old rice seedlings were transplanted artificially on June 17, 2015; June 25, 2016; June 8, 2017 and June 12, 2018 at a row spacing of 25 cm and plant spacing of 20 cm. They were harvested on November 24, 2015; November 24, 2016; November 7, 2017 and October 22, 2018, respectively, before measuring the yields.

Three fertilizer treatments were conducted at the Jiangning experimental station in 2018 (Table 1). (1) CO: control with no fertilization application; (2) CF (Conventional Fertilization): the nutrient application rates were N (255 kg ha^−1^), P_2_O_5_ (64 kg ha^−1^), and K_2_O (76.5 kg ha^−1^). Controlled release fertilizer was used as the basal fertilizer with a top dressing of urea. Nitrogen fertilizer was applied as basal fertilizer, tillering fertilizer and panicle fertilizer with the ratio of 7.5: 1.5: 1; and (3) OF (Optimized Fertilization): the nutrient application rates were N (192 kg ha^−1^), P_2_O_5_ (64 kg ha^−1^), and K_2_O (76.5 kg ha^−1^). Controlled release fertilizer was used as the basal fertilizer. Every treatment was designed with four replicates. Each plot area was 800 m^2^ (8 m × 100 m).

We used the japonica rice cultivar Nanjing 5055 with strong resistance to diseases such as stripe disease, rice smut and sheath blight. The 20-day-old rice seedlings were transplanted by machine on June 16, 2018 at a row spacing of 18 cm and plant spacing of 10 cm. They were harvested on November 9, 2018, and the yield was measured.

### Sampling and laboratory procedures for all experiments

In experiment 1, the leaf samples were collected at tillering, jointing, full panicle and mature growth stages from each experimental plot in the 2015 rice season. In 2016, 2017, and 2018, leaf samples were collected at mature growth stages only. In experiment 2, the leaf samples were collected only at mature growth stage. All plant samples were oven-dried at 105  °C for 30 min and then at 60  °C for 2 days. They were then ground for photoacoustic spectral recording and total N determination. The total nitrogen concentration of leaf samples was determined using a SmartChem 200 discrete auto analyzer (AMS Alliance, Frepillon, France).

### Photoacoustic spectra recording

A FTIR Nicolet 6700 spectrometer (Thermo Electron Scientific Instruments Corporation, Madison, WI, USA) with a model 300 photoacoustic cell (MTEC Photoacoustics, Inc., Oakland, California, USA) was used to record FTIR spectrum of leaf samples. An appropriate amount of ground samples was placed in the photoacoustic cup for direct determination in the infrared photoacoustic spectrum. Before the determination was made, helium was purged for 20 s to reduce the infrared absorption interference from carbon dioxide and water in the air [18]. Scans were conducted in the wavenumber range of 4000 to 500 cm^−1^, the mirror speed was set to 0.32 cm s^−1^, and the resolution was 4 cm^−1^. Each sample was scanned 32 times in succession.

### Data processing

The FTIR-PAS rice spectra were smoothed to reduce noise using the Savitzky–Golay filter [12, 32]. Principal component analysis (PCA) is a multivariate statistical analysis method in which multiple variables are linearly transformed to select fewer important variables [33]. Deconvolution was used to separate the comprehensive information among the spectrum [34, 35]. The objective of deconvolution curve fitting was to separate each peak, which may contain many independent peaks.

The principle are as follows:

1 $$Y\left( x \right) = \varSigma Fi\left( x \right)$$

where *Y, x,* and *i* (1, 2, 3,…*n*) represent the spectrum, wavenumber and number of isolated peak, respectively, the *F* is the expansion function or the kernel function of deconvolution. The Gauss function used was:

2 $$y = \frac{{a_{0} }}{{\pi \sqrt {\pi a_{2} } }}exp\left[ { - \frac{1}{2}\left( {\frac{{x - a_{1} }}{{a_{2} }}} \right)^{2} } \right]$$

where *a*_*0*_*, a*_1_, and *a*_2_ represent the peak amplitude, position, and width, respectively, while *x* and *y* are the wavenumber and photoacoustic intensity respectively.

Preprocessing of photoacoustic spectra and PCA were performed in Matlab R2016a (The MathWorks, Natick, MA, USA). Curvefitting was conducted using Peakfit 4.12. Analysis of variance (ANOVA) was performed using SPSS 19.0 for Windows (SPSS Inc., Chicago, IL, USA).

## Results

### Mid-infrared photoacoustic spectra of leaves

**Fig. 1:**
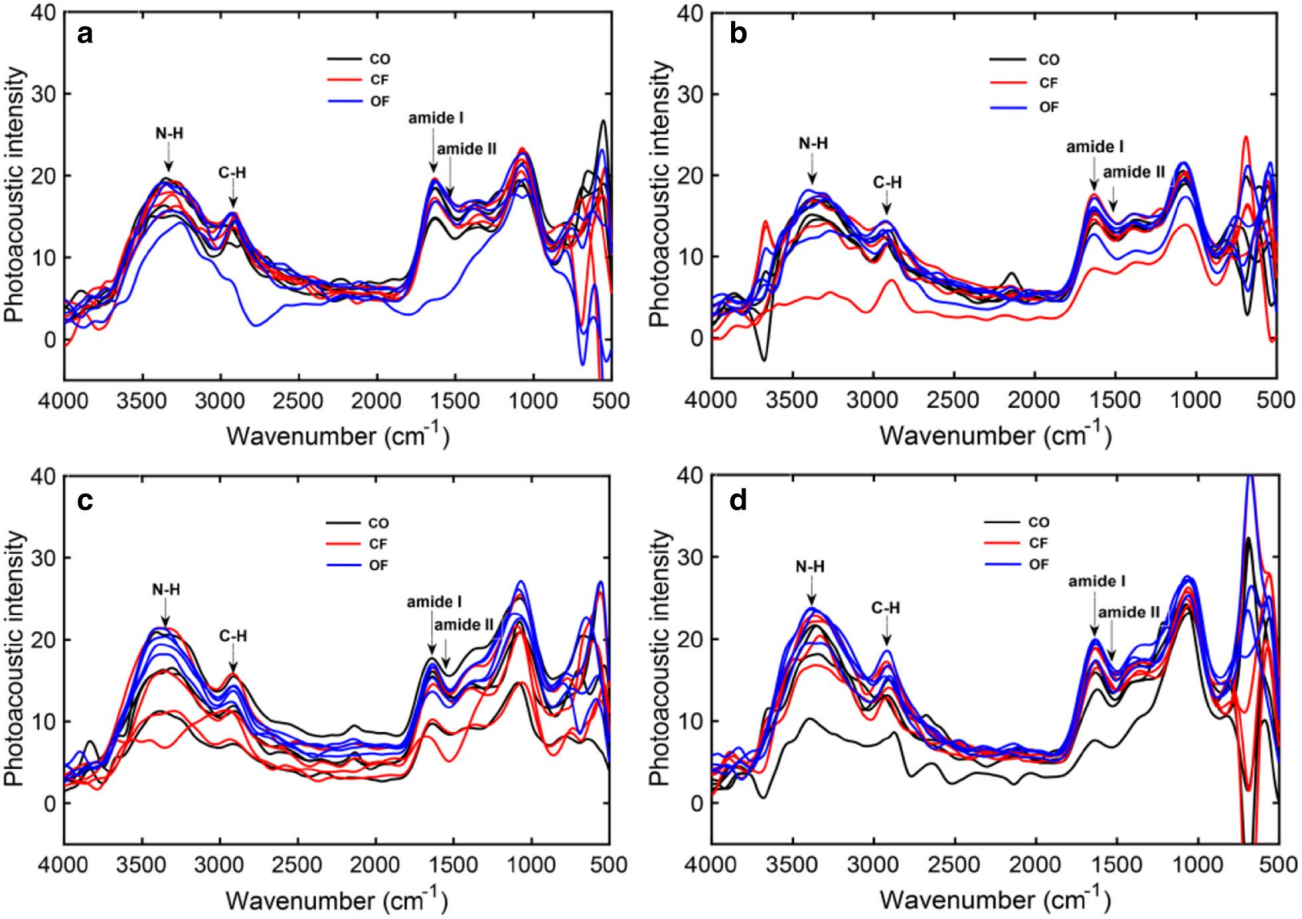
FTIR-PAS of leaves from different Rice stages with different treatments in 2015 growth season. **a** Tillering stage, **b** jointing stage, **c** full panicle stage, **d** mature stage

The photoacoustic spectra of rice leaves from tillering, jointing, full panicle and mature stages with different treatments in 2015 are shown in Fig. 1, and the photoacoustic spectra of rice leaves in 2016, 2017 and 2018 can be obtained from additional file (Additional file 1: Figure S1).

The total spectral shapes and typical bands for all the treatments were similar among the different growth stages. The peak seen at approximately 3365 cm^−1^ was associated with N–H stretching vibration; the peak at 2925 cm^−1^ was associated with C–H stretching vibration [36]; the peak in the range of 1680 to 1630 cm^−1^ was associated with stretching vibration of C=O from amide I, and a small shoulder peak in the range of 1570 to 1510 cm^−1^ was associated with amide II. Spectral intensities of these two bands varied but the band position unchanged. Besides, the fingerprint around 1060 cm^−1^ was caused by C–O stretching vibration. Despite the spectral intensity changes in the wavenumber range of 1570–1510 cm^−1^ and 1680–1630 cm^−1^, the change laws among the different stages and treatments were not found. Further, the principal component analysis (PCA) based on photoacoustic spectra of Rice leaves at the growth stages with different treatments was conducted. However, at each growth stage, the score plot exhibited an irregular pattern among the different treatments (Additional file 1: Figure S2). According to some studies, the full spectrum might contain a lot of disturbed information interferences, which could reduce prediction capacity, robustness, and accuracy [37–39]. Hence, PCA distribution with full spectral range is not appropriate for the assessment of nitrogen status of rice. Therefore, specific wavelength range and other algorithms were considered for achieving the target diagnosis.

### Curve-fitting through deconvolution

**Fig. 2:**
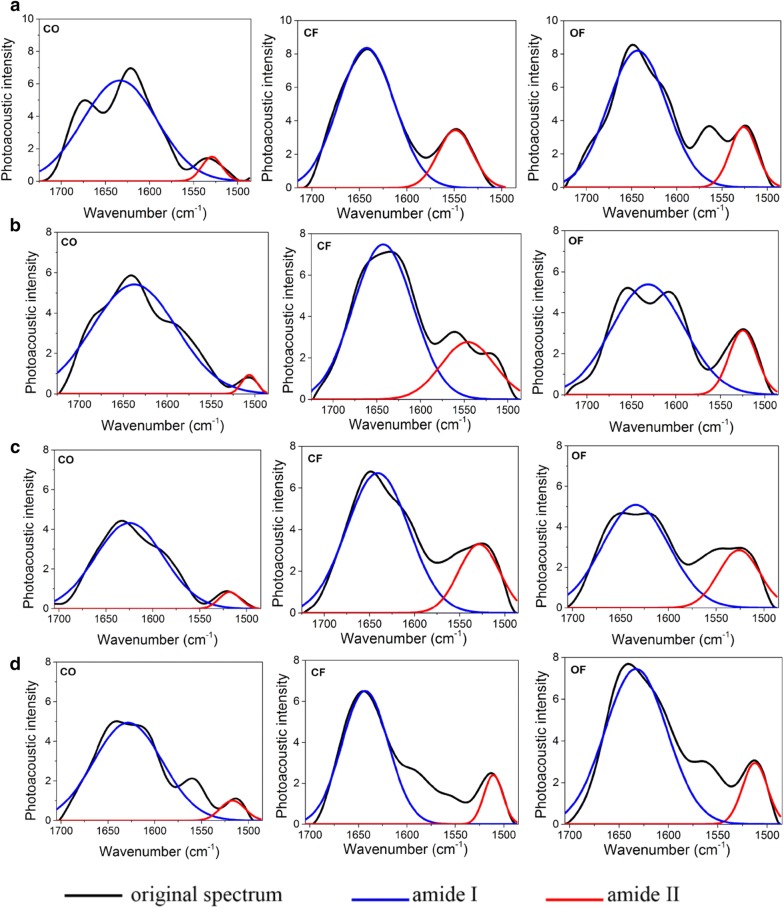
Deconvolution Curve-fitting with different treatments at four stages in 2015 growth season. **a** Tillering stage, **b** jointing stage, **c** full panicle stage, **d** mature stage; *CO* control with no fertilization application, *CF* conventional fertilization, *OF* optimized fertilization

Two peaks indicative of amide among the four growth stages with different treatments in 2015 are shown in Fig. 2. The correlation coefficients (R^2^) between original and fitted spectra were above 0.8 and standard deviations were less than 1. Two peaks appear almost in the same spectral band of different treatments. The first peak related to amide I was at 1680–1630 cm^−1^ and the second peak was nearly at 1570–1510 cm^−1^. Thus, two independent peaks were formed, the former by the stretching vibration of the C=O from amide I and the lateral by the stretching vibration of C–N and the deformation vibration of N–H from amide II [29, 40].

The bands representing amide I and amide II, despite showing approximately similar positions among the different growth stages and treatments, differed in absorption intensity. Amide has been proved to be the main chemical form for Nitrogen storage in plants. Therefore, it was presumed that these two absorption bands could be assessed for nitrogen status in rice. These two peaks were subjected to peak-fitting to find out possible relevance among the growth stages and different treatments. The effect of peak-fitting was determined by the correlation coefficient between fitted and original spectra, and the standard deviation. The higher correlation coefficient (R^2^) resulted in smaller standard deviation as well as better effect of peak-fitting. The deconvolution curve-fitting in 2016, 2017, and 2018 appeared in Additional file 1: Figure S3.

### Diagnosis of nitrogen status in rice

As shown in Table 2, the yield of CO treatment was significantly lower than the other two treatments indicating that no nitrogen supply caused a low yield. In comparison with CF treatment, OF treatment showed a higher yield with an increment of 4.0%–7.8%. There were significant differences between CO and the other two treatments on total nitrogen concentration in rice leaves at the four growth stages in 2015 growth season, whereas no difference was found between CF and OF treatments (Table 3). However, the total nitrogen at mature stage was far less than the other three growth stages, implying that the nitrogen transportation occurred in the growth stages. Besides, for the mature stage from 2016, 2017 and 2018 growth seasons, the differences between CO and the other two treatments on total nitrogen concentration in rice leaves were also significant, whereas there were no significant differences between CF and OF treatments, implying that fertilization can significantly increase the rice leaves nitrogen concentration.

**Table 2:** Effect of N application on rice yield with different treatments in different growth seasons

Experimental station	Seasons	Rice yield (kg ha^−1^)		
		CO	CF	OF
Tangquan	2015	7575 ± 282.2^b^	9432 ± 345.6^a^	9872 ± 297.5^a^
	2016	6385 ± 255.5^b^	8633 ± 246.3^a^	9155 ± 392.5^a^
	2017	4095 ± 195.7^b^	5844 ± 246.8^a^	6297 ± 268.5^a^
	2018	6887 ± 323.7^b^	8925 ± 267.5^a^	9375 ± 422.9^a^
Jiangning	2018	7212 ± 258.6^b^	11077 ± 532.7^a^	11515 ± 478.7^a^

Data are expressed as the means of four replications. Significant differences (*P* ≤ 0.05) among treatments within each line are indicated with different letters

**Table 3:** Effect of N application on total N concentration of rice leaves with different treatments in different growth seasons

Experimental station	Seasons	Treatments	Total N (g kg^−1^)			
			Tillering stage	Jointing stage	Panicle stage	Mature stage
Tangquan		CO	10.58 ± 0.52^b^	9.95 ± 0.37^b^	10.52 ± 0.55^b^	9.97 ± 0.47^b^
	2015	CF	24.98 ± 0.75^a^	22.00 ± 0.59^a^	29.11 ± 0.77^a^	12.45 ± 0.35^a^
		OF	28.65 ± 0.68^a^	26.61 ± 0.85^a^	25.13 ± 0.65^a^	12.05 ± 0.38^a^
		CO	–	–	–	10.49 ± 0.20^b^
	2016	CF	–	–	–	12.31 ± 0.30^a^
		OF	–	–	–	12.40 ± 0.55^a^
		CO	–	–	–	10.23 ± 0.26^b^
	2017	CF	–	–	–	11.87 ± 0.65^a^
		OF	–	–	–	11.90 ± 0.46^a^
		CO	–	–	–	9.86 ± 0.0.57^b^
	2018	CF	–	–	–	11.81 ± 0.44^a^
		OF	–	–	–	11.09 ± 0.25
Jiangning	2018	CO	–	–	–	9.51 ± 0.32^b^
		CF	–	–	–	11.28 ± 0.36^a^
		OF	–	–	–	10.68 ± 0.35^a^

Data are expressed as the means of four replications. Significant differences (P ≤ 0.05) among treatments within each column are indicated with different letters

**Fig. 3:**
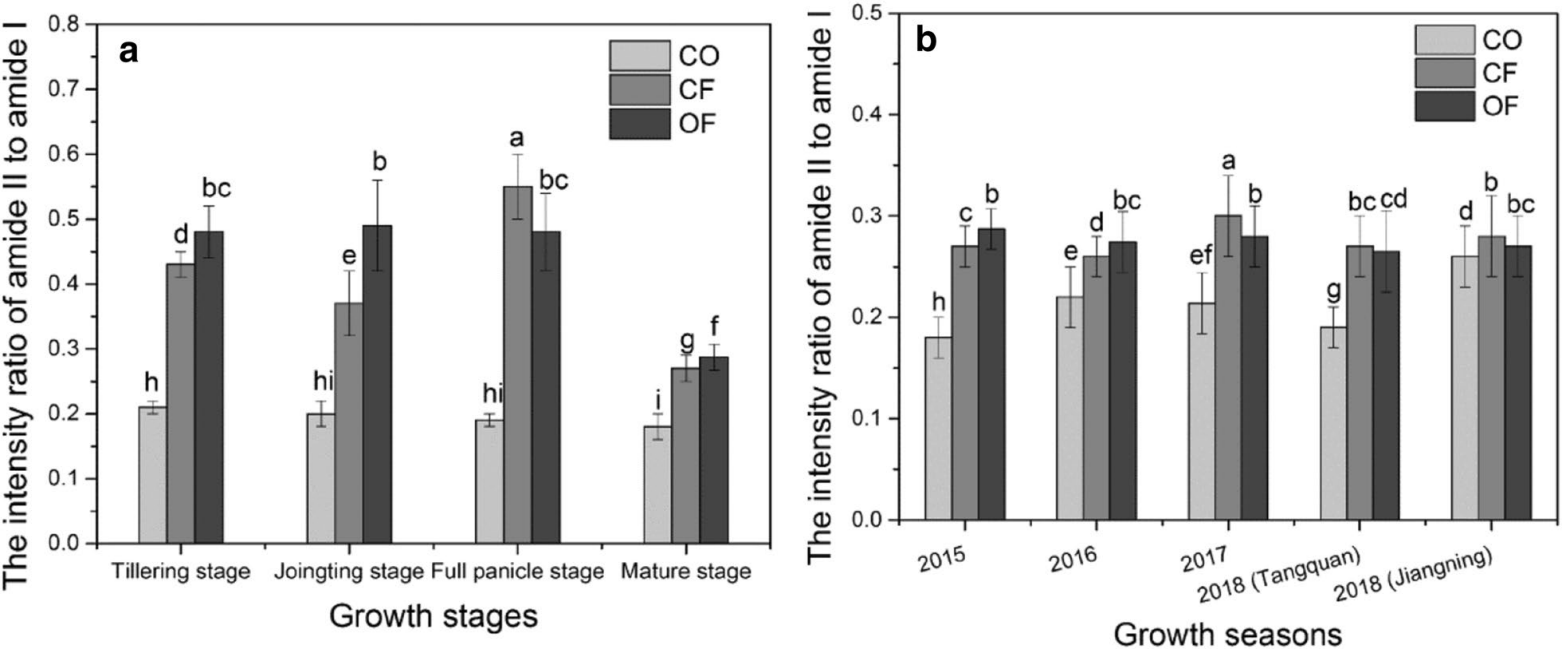
The photoacoustic intensity ratio of amide II to amide I. CO Control with no fertilization application, CF Conventional Fertilization, OF Optimized Fertilization. **a** The ratio of amide II to amide I in different treatments at four growth stages in the 2015 growth season, **b** the ratio of amide II to amide I in different treatments at mature stage in the 2015, 2016, 2017 and 2018 growth seasons. Bars are the standard error of the means. Different letters above the bars indicate a significant difference at P < 0.05 according to the Duncan test

In the spectrum, the intensity of spectral absorption was usually interfered by the instrument and external environment [18]. Therefore, the ratio of photoacoustic intensity of amide II to amide I in rice leaves was recorded to avoid this interference (Fig. 3). In 2015 growth season, the ratios of photoacoustic intensity of amide II to amide I in rice leaves sampled in CF and OF treatments were significantly higher than the ratio of CO treatment (Fig. 3a). At tillering stage, the ratioof CO treatment showed a minimal value of 0.21, however, the same ratio reached 0.43 and 0.48 when CF and OF treatments were used, respectively. Subsequently, at the Jointing stage, the ratio reached 0.49, which was higher than that of CO and CF treatments. At full panicle stage, the ratio of OF treatment was still at a high level (0.48) due to the continuously release nitrogen nutrition from coated urea, whereas the ratio reached 0.55 when CF treatment was used, because of the top dressing fertilization with urea, thus providing sufficient nitrogen nutrition for rice, resulting in a higher content of nitrogen in the sampled leaves. The ratio decreased at the mature stage compared to that at the full panicle stage regardless of the treatment used. Related research shows that the primary and secondary amides are the dominant forms of nitrogen in plants [27]. When supplied with excess nitrogen, extra nitrogen was transformed into secondary amides, therefore, In CO, with no-nitrogen supply, the ratio (approximately 0.2) was much lower than in CF or OF. At the later growth stages, the grain absorbs nearly 80% of the nitrogen found especially in leaves and in stems [24]. Thus, two-thirds of the nitrogen in stems and leaves was transported to the ears for grain filling [41]. Due to the application of coated urea (CO(NH_2_)_2_)at the first three stages, the ratio in OF was relatively stable (above 0.48), that is, the ability of the slow-releasing coated urea to supply nitrogen to the plants was relatively long lasting.

**Fig. 4:**
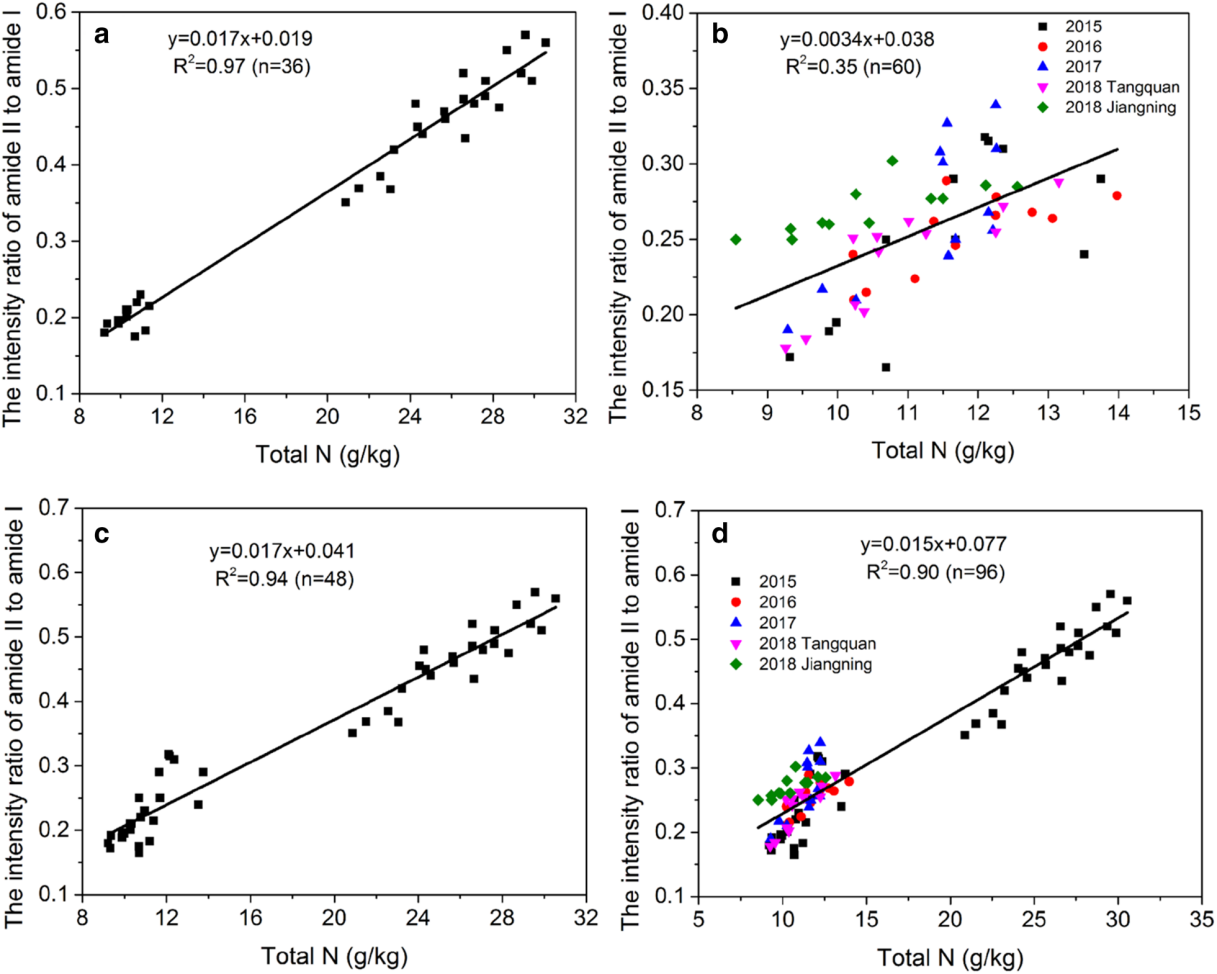
Linear regression between the total N concentration of rice leaves and the ratio of amide II to amide I. **a** The 2015 growth season for the first three stages only excluding the mature stage. **b** Only at the maturity from all growth seasons. **c** All growth stages in the 2015 season. **d** 2015, 2016, 2017 and 2018 growth seasons. The linear functions and the square of the Correlation Coefficient (R^2^) are shown in the each plot

Overall, increased nitrogen accumulation in stems and leaves led to increased nitrogen transportation for grain filling and thereby increased the yield [42, 43]. Therefore, the nitrogen status of the leaves can be used to characterize the nitrogen nutritional status of rice. In addition, at the first three growth stages in 2015, the total nitrogen concentration of leaves showed a positively linear correlation (R^2^ = 0.97) with the intensity ratio of amide II to amide I (Fig. 4a). In 2015, 2016, 2017 and 2018 mature stages, the ratio of CO was significantly lower than the ratio of CF and OF treatments. For all treatments, the ratios were below 0.3 (Fig. 3b) and displayed relatively low relevance (R^2^ = 0.35) between the ratio and total nitrogen concentration (Fig. 4b). Besides, for the maturity from every growth season, the correlation still remained relatively low (Additional file 1: Figure S4), which can probably be due to the fact that only mature leaf samples were measured. However, in 2015 growth season, the R^2^ was 0.94 (Fig. 4c), while for all the growth seasons, the R^2^ reached 0.9 (Fig. 4d), implying a good correlation between the total N and the ratio. Therefore, our results suggest that the intensity ratio of amide II to amide I can reflect the total N of the rice leaves well through the growth stages.

Since tillering and grouting play an important role in rice yield [22, 44]. Thus, the ratio at the tillering and full panicle stages were more suitable for diagnosis. At these two stages, the ratios of CF and OF treatments were above 0.4, moreover, at full panicle stage, the ratio of CF treatment reached 0.55; whereas the ratios of CO (with no fertilization) were around 0.2. Therefore, taking into account the level and stages of nitrogen supply among the different treatments, the intensity ratio of amide II to amide I in rice leaves, and combined with the rice yield, a ratio of 0.4–0.55 reflects a suitable plant nitrogen status. When the ratio is less than 0.4, nitrogen content in the plant leaf tissues can be considered insufficient, while a ratio above 0.55 implies excess nitrogen in the plant tissues.

## Discussion

Our study shows that the correlation between the intensity ratio and rice leaves total Nitrogen concentration was relatively high with a coefficient of 0.94 throughout the 2015 growth season (Fig. 4c). However, the correlation coefficient (R^2^) at mature stage from 2015, 2016, 2017 and 2018 growth seasons was only 0.36 (Fig. 4b), similarly, the correlations were also not relatively high for the mature stage from every growth season (Additional file 1: Figure S4). This is mainly due to the fact that the total N concentration and ratio in leaves at mature stage were relatively low, and the variations of total N concentration and ratio were slighter compared with the calculation throughout the growth season, thus resulting in a relatively not high correlation. Besides, for the mature stage from all growth seasons, the ratios were below 0.3 among different treatments.

In 2018, at Jiangning station, the ratio of CO was very close to the ratios of CF and OF, implying that the ratio was poorly respond to the Nitrogen supply. Therefore, the ratio at the mature stage was not suitable for nitrogen diagnosis. Meanwhile, according to the analysis of all data samples in four growth seasons, the intensity ratio of amide II to amide I and the total Nitrogen concentration still showed a high correlation (Fig. 4d). This still indicates that during the entire growth period of Rice, the ratio can be used to assess the Nitrogen levels. In our study, at the tillering and full panicle stages, a ratio of 0.4–0.55 was considered appropriate. However, for Chinese cabbage, the ratio of 0.9–1.0 was suitable for its growth and without massive Nitrate accumulation [18], indicating that the appropriate ratio depends on the plant species. In addition, in our field experiments, we used three rice cultivars, whether this ratio is applicable to all rice cultivars is still unknown. Therefore, more cultivars need to be studied in future to increase the scope of this experiment.

## Conclusions

According to the FTIR-PAS of rice leaves, the ratio of photoacoustic intensity of amide II to amide I was positively correlated to different nitrogen treatments and growth stages, and the ratios at tillering and full panicle stages were more suitable for nitrogen diagnosis. A ratio of 0.4–0.55 likely reflects a suitable nitrogen status for rice. For a ratio less than 0.4, the nitrogen status can be considered insufficient, whereas a ratio above 0.55 implies excess nitrogen. Thus, by using the FTIR-PAS, we can facilitate effective assessment of the nitrogen status in rice production, which can provide appropriate guidance for rational fertilization.

## Additional file

**Additional file 1.** Additional figures.

## Data Availability

The datasets supporting the conclusion of this article are included within the article (additional files).
