# Supplementary material for: Rapid diagnosis of nitrogen status in rice based on Fourier transform infrared photoacoustic spectroscopy (FTIR-PAS)
Source: Plant Methods. 2019 Aug 19;15:94. doi: 10.1186/s13007-019-0482-0 (PMC6699123; doi:10.1186/s13007-019-0482-0)
Supplement: Supplementary file 1 — Additional file 1. Additional figures. [file 13007_2019_482_MOESM1_ESM.docx]

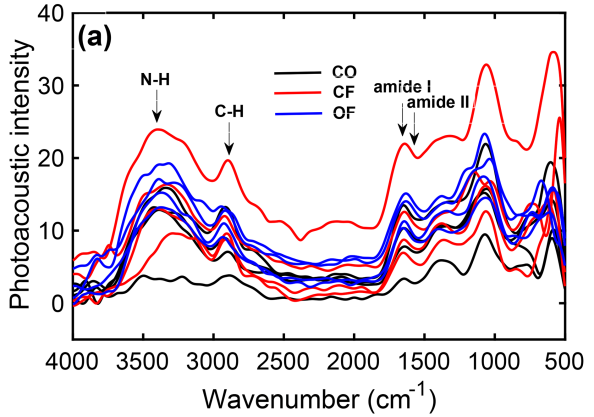

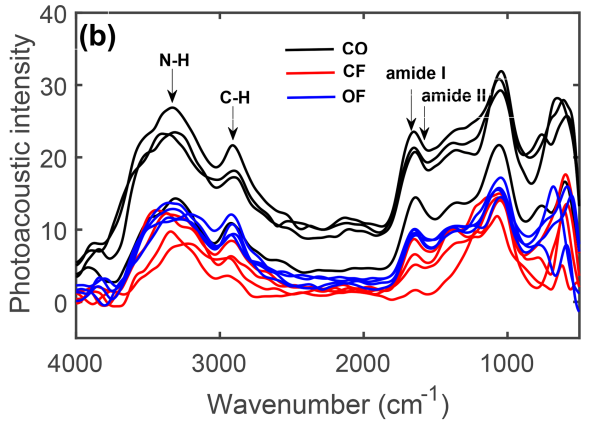


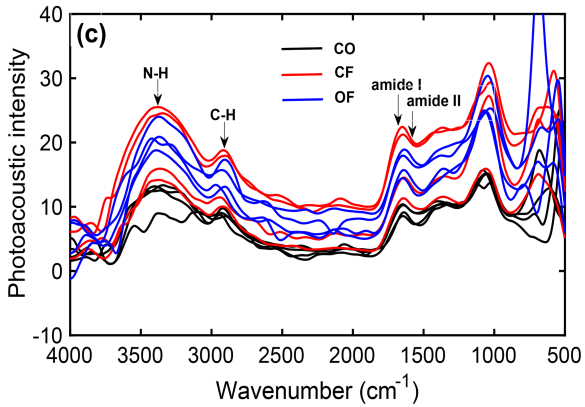

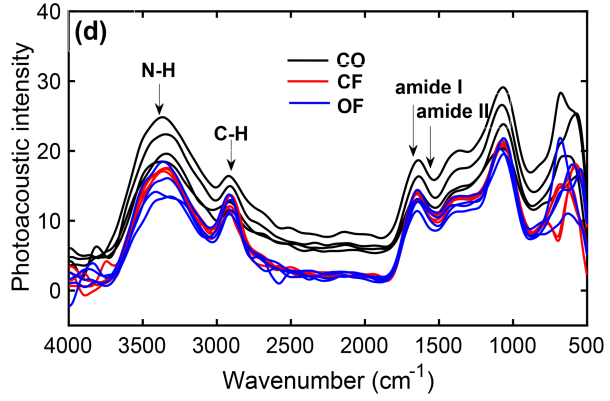


**Figure S1** FTIR-PAS spectra of rice leaves at mature stage in 2016, 2017 and 2018 growth seasons. **a.** 2016 growth season. **b.** 2017 growth season. **c.** 2018 growth season at Tangquan experimental station. **d.** 2018 growth season at Jiangning experimental station.


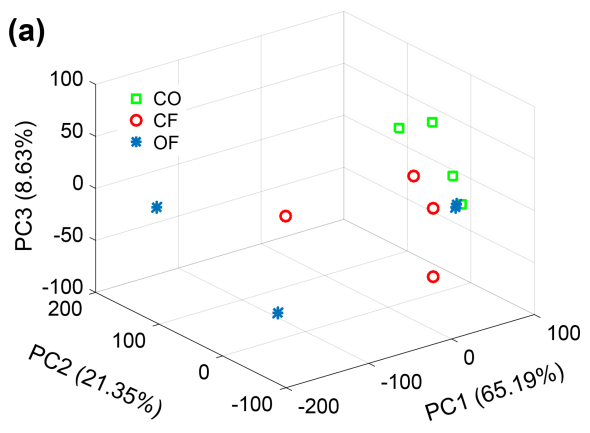

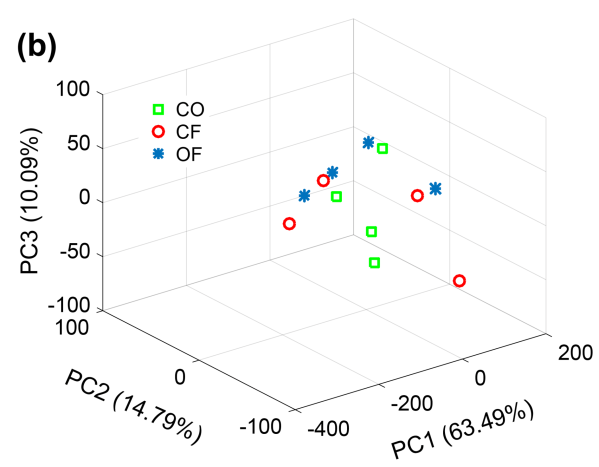


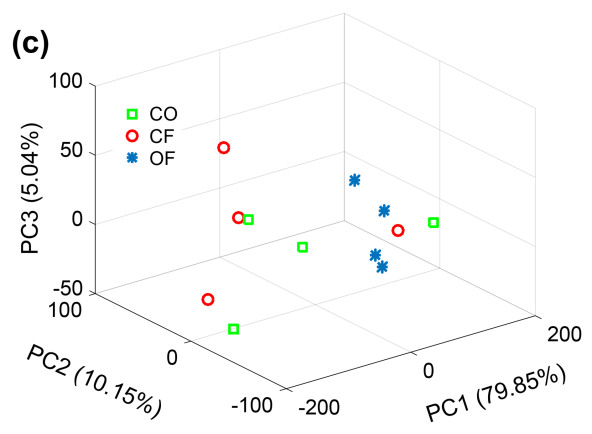

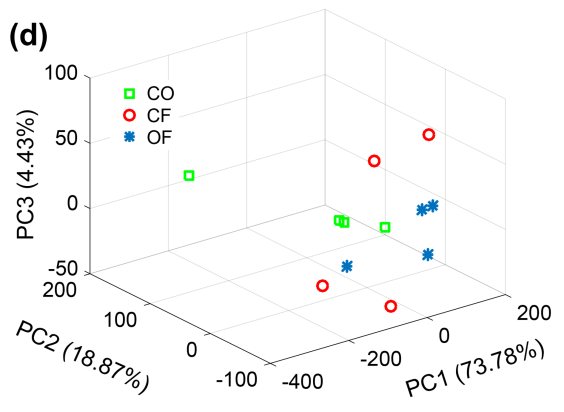


**Figure S2** Principal component distributions based on FTIR-PAS from different stages in different treatments. The explained variances of the first three principal components PC1, PC2 and PC3 are shown in brackets. (a) Tillering stage, (b) Jointing stage, (c) Full panicle stage, (d) Mature stage.

Original spectrum amide I amide II

**a**









**b**









**c**









**d**









**Figure S3** Deconvolution curve-fitting of mature stage in 2016, 2017 and 2018 growth seasons. **a.** 2016 growth season. **b.** 2017 growth season. **c.** 2018 growth season at Tangquan experimental station. **d.** 2018 growth season at Jiangning experimental station.















**Figure S4** Linear regression between the total N concentration of rice leaves and the ratio of amide II to amide I at mature stage. (a) 2015 growth season. (b) 2016 growth season. (c) 2017 growth season. (d) 2018 growth season at Tangquan station. (e) 2018 growth season at Jiangning station.
